# Hemophagocytic lymphohistiocytosis in kidney transplantation: a call for heightened vigilance

**DOI:** 10.1007/s40620-025-02446-8

**Published:** 2025-12-04

**Authors:** Ahmed Mansour Abdel-Rahman, Muhammed Ahmed Elhadedy, Donia Elsaed Gad, Sara Elsherbini, Ayman F. Refaie

**Affiliations:** 1 Dialysis and Transplantation Unit, Urology and Nephrology Center, Mansoura University, Mansoura, Egypt; 2 Pathology Department, Faculty of Medicine, Mansoura University, Mansoura, Egypt

**Keywords:** Hemophagocytic lymphohistiocytosis, Kidney transplantation, Fever, Hyperferritinemia

## Abstract

Hemophagocytic lymphohistiocytosis is a rare, severe, and potentially fatal disease, mainly encountered in individuals with immunologic diseases. Early diagnosis and prompt intervention may be life-saving. Herein, we report a case of a 52-year-old man who had undergone kidney transplantation nine years previously, presenting with persistent fever of unknown origin and graft function impairment. Despite receiving broad-spectrum antimicrobials and undergoing an exhaustive work-up for infectious, autoimmune, and oncologic diseases, he developed pancytopenia, hepatosplenomegaly, and respiratory failure. A bone marrow biopsy revealed florid hemophagocytosis, hyperferritinemia, hypertriglyceridemia, elevated serum soluble CD25, and cytopenia. These findings fulfilled seven of the Hemophagocytic lymphohistiocytosis 2004 criteria. High dose dexamethasone induced a partial, transitory response and lowered the fever, but multiorgan failure progressed, and the patient died three weeks after ICU admission. This case underscores that hemophagocytic lymphohistiocytosis should be considered whenever kidney transplant recipients develop unexplained fever, cytopenia, and hyperferritinemia, and suggests that early bone marrow evaluation with targeted immunomodulation may improve outcomes in this vulnerable population.

**Graphical Abstract:**

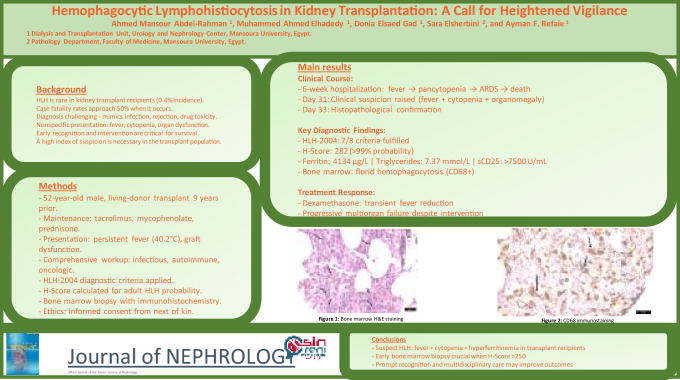

## Introduction

Hemophagocytic lymphohistiocytosis is a rare, life-threatening hyperinflammatory syndrome characterized by dysregulated immune activation and multi-organ dysfunction. Initially described in pediatric patients, it is increasingly recognized in adults, with reported mortality rates ranging from 26 to 75% [1, 2]. Hemophagocytic lymphohistiocytosis is categorized as primary (familial) or secondary (acquired); secondary cases are usually triggered by infections, malignancies, or autoimmune disorders [2].

Secondary hemophagocytic lymphohistiocytosis is a particular concern in solid organ transplant recipients, where the risk is increased due to chronic immunosuppression. In kidney transplant patients, hemophagocytic lymphohistiocytosis is rare; one single-center analysis estimated an incidence of around 0.4% in this population [3]. But when it occurs, it can be devastating, with case fatality rates approaching 50% [4]. The pathophysiology involves impaired cytotoxic function of natural killer (NK) cells and CD8 + T lymphocytes, leading to unchecked macrophage activation and hemophagocytosis, resulting in an overwhelming cytokine release with tissue damage [5].

Diagnosing hemophagocytic lymphohistiocytosis in transplant recipients is challenging, as the presentation is nonspecific and may mimic common post-transplant complications such as chronic infection, acute rejection, or drug toxicity. The Hemophagocytic lymphohistiocytosis-2004 diagnostic criteria (developed in pediatrics) and the newer HScore (an adult scoring system for secondary reactive hemophagocytic lymphohistiocytosis) are commonly used diagnostic tools [6, 7]. Confirmation often requires histopathological evidence of hemophagocytosis on bone marrow examination, along with the demonstration of characteristic laboratory abnormalities including cytopenias, hyperferritinemia, hypertriglyceridemia, and elevated soluble interleukin-2 receptor (sCD25) levels [6].

No standardized treatment guidelines exist for hemophagocytic lymphohistiocytosis, and this also applies to kidney transplant recipients. Management typically involves a multitargeted approach: addressing the inciting trigger, adjusting or reducing baseline immunosuppression, administering high-dose corticosteroids, and in severe cases initiating chemotherapy based on Hemophagocytic lymphohistiocytosis-94/2004 protocols [8]. Early recognition and immediate intervention are critical to improving outcomes in this setting.

Here, we present a 52-year-old kidney transplant recipient with persistent fever of unknown origin who was ultimately diagnosed with hemophagocytic lymphohistiocytosis, underscoring the diagnostic challenges, therapeutic obstacles, and need for a high index of suspicion for this rare, but potentially fatal condition.

## Case report

### Clinical presentation (hospital day 0)

A 52-year-old male, who had received a living-donor kidney transplant nine years previously, presented with fever (38.5 °C), sore throat, and mild graft dysfunction (serum creatinine 0.9 → 1.2 mg/dL). His maintenance immunosuppression comprised tacrolimus, mycophenolate mofetil (1.5 gm/day), and low dose prednisone (5 mg/day). He had a history of bleeding hemorrhoids for which he had declined surgical treatment two months earlier; this was initially considered a plausible explanation for his normocytic anemia (hemoglobin [Hb] 8.5 g/dL). Physical examination was otherwise unremarkable. Baseline platelet levels were 164 × 10⁹/L, white blood cells (WBC) 11 × 10⁹/L, and tacrolimus trough level 6.2 ng/mL. Computed tomography (CT) scan of the chest, abdomen, and pelvis on presentation revealed no pulmonary infiltrates, lymphadenopathy, or graft abnormality.

### Initial management (hospital days 1–30)

Ampicillin–sulbactam was started empirically, but fever persisted. On hospital day 5, antimicrobial therapy was broadened to meropenem despite persistently sterile blood and urine cultures. This escalation was justified by the progressive rise in both C-reactive protein and procalcitonin, and the low sensitivity of routine cultures for detecting occult infections in transplant recipients. Mycophenolate mofetil was therefore withheld to minimize additional immunosuppressive pressure during this period of diagnostic uncertainty. Nuclear magnetic resonance imaging (MRI) of the abdomen and pelvis on day 10 showed mild hepatosplenomegaly with a structurally normal renal allograft; upper and lower gastrointestinal endoscopies were normal. We interpreted this hepatosplenomegaly as a reactive enlargement secondary to the ongoing systemic inflammatory process. A follow-up abdominal ultrasound on Day 14 confirmed unchanged hepatosplenomegaly with normal parenchymal echotexture and no focal lesions, supporting a reactive rather than obstructive or infiltrative cause. At this stage, triglycerides and ferritin had not yet been measured. Routine surveillance included total cholesterol only, and the early anemia was attributed to chronic hemorrhoids.

Comprehensive infectious screening—including acid-fast bacillus stain and PCR, QuantiFERON-TB assay, COVID-19 PCR, viral hepatitis serology, Widal test, and Brucella IgG—was negative. In parallel, quantitative CMV-DNA and EBV-DNA PCRs performed on peripheral blood were both < 200 IU mL⁻1, effectively ruling out active viremia. Bone-marrow samples were therefore reserved for cytological analysis rather than repeat viral testing. Autoimmune panel (ANA, anti-dsDNA) and neoplastic work-up (alpha-fetoprotein, carcinoembryonic antigen, CA19-9, PSA) gave no diagnostic clues. Likewise, serum protein electrophoresis and kappa/lambda ratio were within normal limits. As fever > 39 °C persisted, additional antimicrobials (levofloxacin, vancomycin, linezolid, acyclovir, oseltamivir, fluconazole) were introduced sequentially. Anemia worsened (nadir Hb 7.0 g/dL), necessitating three packed red blood cell (RBC) transfusions, although testing for occult gastrointestinal bleeding and hemolysis were negative. By Day 25, the patient developed pancytopenia (WBC 2.9 × 10⁹/L; platelets 75 × 10⁹/L). At each stage we also ruled out acute rejection, drug toxicity, thrombotic microangiopathy, post-transplant lymphoproliferative disorder, and occult viral infections such as cytomegalovirus (CMV) before hemophagocytic lymphohistiocytosis emerged as the most likely explanation.

### Diagnosis of hemophagocytic lymphohistiocytosis (hospital days 31–33)

**Fig. 1:**
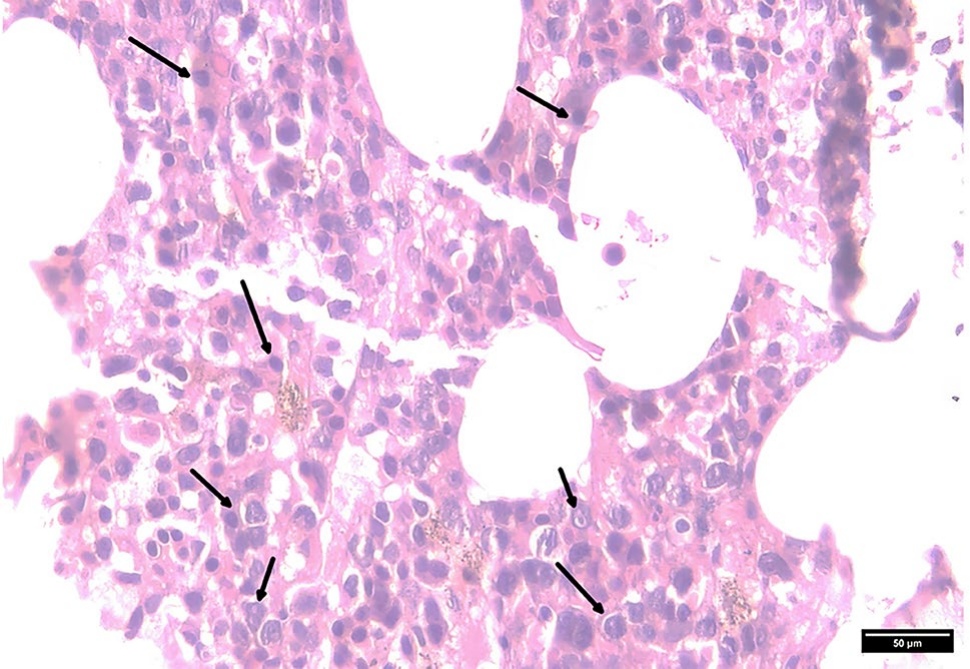
Bone marrow histopathology demonstrating hemophagocytosis. Bone marrow biopsy showing large lymphoid aggregates with associated hemophagocytosis (arrows). Note the histiocytes actively engulfing multiple hematopoietic cells, characteristic of hemophagocytic lymphohistiocytosis. (Hematoxylin & Eosin, × 400)

**Fig. 2:**
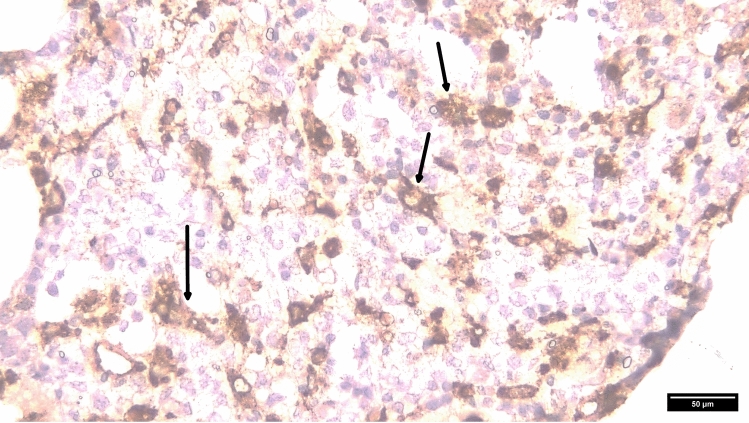
Immunohistochemical confirmation of hemophagocytic activity. Immunohistochemical staining for CD68 highlights the increased number of activated histiocytes with evident hemophagocytic activity. This confirms the macrophage lineage of the phagocytic cells. (CD68, × 400)

By Day 31, the combination of persistent fever without a clear cause, new hepatosplenomegaly, and worsening pancytopenia raised a strong clinical suspicion of secondary hemophagocytic lymphohistiocytosis. A bedside bone-marrow aspiration was attempted on Day 29 when platelets were 78 × 10⁹/L; however, the specimen was hypocellular and non-diagnostic. The same day, the patient developed acute hypoxic respiratory failure; chest CT revealed bilateral infiltrates consistent with acute respiratory distress syndrome (ARDS), necessitating continuous positive airway pressure (CPAP). Because the patient then developed ARDS with labile blood pressure, and platelets dropped to 75 × 10⁹/L, a repeat, image-guided marrow biopsy was deferred until hemodynamic and coagulation parameters were stabilized. Dexamethasone 8 mg every 12 h was administered on Day 32, and his temperature improved after two doses, supporting an inflammatory etiology. Triglycerides and ferritin were first checked on Day 29, when hemophagocytic lymphohistiocytosis was suspected, and were markedly elevated (see Table 2). Targeted laboratory tests showed marked hypertriglyceridemia (7.37 mmol/L), severe hyperferritinemia (4134 μg/L), and very high serum soluble CD25 (> 7500 U/mL), thereby meeting five of the Hemophagocytic lymphohistiocytosis-2004 criteria (Table 1) [6]. The calculated HScore was 273, corresponding to *a* > 99% probability of hemophagocytic lymphohistiocytosis (Table 2) [7]. Once platelets were corrected with transfusions, a bone marrow biopsy was performed on Day 33, which demonstrated large lymphoid aggregates and florid hemophagocytosis (Fig. 1); CD68 staining confirmed activated histiocytes engulfing blood cells (Fig. 2). With seven diagnostic criteria fulfilled, secondary hemophagocytic lymphohistiocytosis was confirmed. Although his fever eased briefly, multiorgan failure nevertheless progressed.

**Table 1:** Hemophagocytic lymphohistiocytosis-2004 Diagnostic Criteria findings in Our Patient

HLH-2004 Diagnostic Criteria	Findings in Our Patient	Status
Fever ≥ 38.5 °C	Maximum 40.2 °C	Present
Splenomegaly	Mild splenomegaly on MRI	Present
Cytopenias affecting ≥ 2 lineages • Hb < 90 g L⁻^1^ → 85 g/L • Platelets < 100 × 10⁹ /L → 75 × 10⁹ /L	Two cytopenic lineages documented; neutrophil count not below 1.0 × 10⁹ L⁻^1^	Present
Hypertriglyceridemic ≥ 3.0 mmol/L and/or hypofibrinogenemia ≤ 1.5 g/L	Fasting triglycerides 7.37 mmol/L	Present
Hemophagocytosis in bone marrow, spleen, lymph node, or liver	Bone-marrow biopsy with hemophagocytosis (CD68 +)	Present
Low or absent natural killer cell activity	Not tested	Not assessed
Ferritin ≥ 500 μg/L	4134 μg/L	Present
Soluble CD25 ≥ 2400 U/mL	> 7500 U/mL	Present

Diagnostic summary. The patient fulfills 7 of 8 HLH-2004 criteria (≥ 5 required), supporting a clinical diagnosis of hemophagocytic lymphohistiocytosis

**Table 2:** H-Score Components in Our Patient

H-Score Parameter	Findings (SI units)	Points
Maximum temperature, °C	40.2	49
Organomegaly	Hepatomegaly + splenomegaly (MRI)	38
Cytopenias (number of lineages)	Two lineages affected (Hb 85 g/L; platelets 75 × 10⁹ /L)	24
Triglycerides, mmol/L	7.37 (≥ 3.0)	64
Fibrinogen, g/L	Not measured	0
Ferritin, μg/L	4134 (2000—6000)	35
AST, IU/L	1435	19
Hemophagocytosis on bone marrow aspirate	Present	35
Known underlying immunosuppression	Kidney transplant recipient on immunosuppressive therapy	18
Total H-Score		282

An H-Score ≥ 250 corresponds to a > 99% post-test probability of hemophagocytic lymphohistiocytosis (HLH)

### Clinical outcome (hospital days 34–45)

Over the twelve days that followed the hemophagocytic lymphohistiocytosis diagnosis, the patient’s respiratory failure worsened, necessitating invasive mechanical ventilation. Despite vasopressor support, broad-spectrum antibiotics, and the best available supportive measures, he developed Klebsiella pneumoniae bacteremia and disseminated intravascular coagulation. Multiorgan failure could not be reversed, and the patient died nearly six weeks after his initial presentation.

## Discussion

This case highlights the complex diagnostic and therapeutic challenges of hemophagocytic lymphohistiocytosis among kidney transplant recipients—a setting where clinical caution is crucial, yet the condition remains under-recognized.

Hemophagocytic lymphohistiocytosis is a hyperinflammatory syndrome that can present with nonspecific symptoms such as fever, cytopenia, and organ dysfunction, features common to many post-transplant complications. In kidney transplant recipients, these signs initially raise suspicion of infection, rejection, or drug toxicity, delaying recognition of hemophagocytic lymphohistiocytosis. Thrombotic microangiopathy, post-transplant lymphoproliferative disorder, and occult infections such as cytomegalovirus can present similarly and must be ruled out early [5, 9]. Moreover, hemophagocytic lymphohistiocytosis itself may contribute to tissue injury that confounds interpretation, including renal dysfunction from acute tubular injury or glomerular microangiopathy [10].

In such scenarios, the HScore offers a helpful diagnostic framework. It integrates clinical and laboratory features—including fever, cytopenias, organomegaly, ferritin, triglycerides, and serum soluble CD25—to estimate the probability of reactive hemophagocytic lymphohistiocytosis in adults. An HScore > 250 carries a sensitivity exceeding 90% [7]. Although the Hemophagocytic lymphohistiocytosis-2004 criteria remain the standard for diagnosis, they were initially developed for pediatric populations and may not capture the full spectrum of hemophagocytic lymphohistiocytosis in adults, particularly in immunosuppressed patients [6]. In our case, the diagnosis became apparent only after seven criteria were met; however, earlier application of the HScore could have supported the diagnosis at an earlier stage. Importantly, bone marrow examination should not be delayed in patients with unexplained fever and cytopenias, particularly when ferritin is markedly elevated.

Hemophagocytic lymphohistiocytosis care in solid-organ transplant recipients is delicate: tapering immunosuppression aids immune recovery and infection control, yet stopping the cytokine storm demands higher doses. Escalation heightens infection risk, whereas delay invites irreversible organ damage [3, 11].

Standard protocols based on Hemophagocytic lymphohistiocytosis-94 and Hemophagocytic lymphohistiocytosis-2004 recommend the use of corticosteroids, etoposide, and cyclosporine A. However, these regimens are rarely adopted in transplant patients due to concerns about toxicity and further immunosuppression [6]. In our patient, high-dose dexamethasone provided transient improvement, but progression to multiorgan failure ensued. Mycophenolate was withheld; however, this intervention alone proved insufficient. There is currently no consensus on how aggressively to treat hemophagocytic lymphohistiocytosis in the transplant population. Case reports have described successful outcomes with steroids alone or in combination with intravenous immunoglobulin (IVIG) and plasma exchange, particularly when an apparent infectious trigger is addressed [3, 4]. More recently, targeted therapies—such as anti-interleukin-1 and Janus kinase inhibitors—have shown promise in refractory hemophagocytic lymphohistiocytosis but remain largely experimental in transplant recipients [11].

Our experience emphasizes that early, decisive therapy, tailored to address both the underlying trigger and the severity of inflammation, is essential. Equally essential is collaboration among transplant specialists, hematologists, and intensivists to weigh the risks and benefits of each therapeutic step.

Hemophagocytic lymphohistiocytosis in transplant recipients is paradoxical: even under chronic immunosuppression to prevent rejection, these individuals remain susceptible to uncontrolled immune activation. Hemophagocytic lymphohistiocytosis represents a breakdown in cytotoxic T-cell and NK-cell regulation, leading to persistent activation of macrophages and overwhelming cytokine release [11, 12]. Immunosuppressive agents, particularly calcineurin inhibitors, do not directly suppress this macrophage-driven process and may impair the clearance of viral or malignant triggers, allowing hemophagocytic lymphohistiocytosis to evolve unchecked [12].

Infections, especially from herpesviruses such as Epstein–Barr virus and CMV, are among the most frequent precipitating factors in transplant-associated hemophagocytic lymphohistiocytosis [13]. These pathogens not only evade immunosurveillance but also trigger dysregulated immune activation. Moreover, the immunocompromised state can mask typical signs of systemic inflammation, making hemophagocytic lymphohistiocytosis harder to detect until organ failure is imminent.

Timely recognition of hemophagocytic lymphohistiocytosis in transplant recipients is critical: unexplained fever, cytopenias, and elevated ferritin, even under immunosuppression, should trigger high suspicion, as early immunomodulatory therapy can prevent irreversible organ failure and death. This case shows that a non-diagnostic early marrow sample can give clinicians false reassurance; arranging a timely, well-prepared repeat biopsy can be decisive.

## Data Availability

The clinical and histopathological data supporting this case report are not openly available due to patient privacy and ethical considerations and are available from the corresponding author upon reasonable request. Data are located in controlled access data storage at the Urology and Nephrology Center, Mansoura University.

## Abbreviations

ANA: Antinuclear antibody
ARDS: Acute respiratory distress syndrome
AST: Aspartate aminotransferase
CA19: Carbohydrate antigen 19-9
CD25: Cluster of differentiation 25 (IL-2 receptor α chain)
CD68: Cluster of differentiation 68 (macrophage marker)
CD8: Cluster of differentiation 8 (cytotoxic T-lymphocyte marker)
CMV: Cytomegalovirus
CMV-DNA: Cytomegalovirus deoxyribonucleic acid
COVID: Coronavirus disease 2019
CPAP: Continuous positive airway pressure
CT: Computed tomography
EBV-DNA: Epstein–Barr virus deoxyribonucleic acid
H-Score: Hemophagocytic syndrome diagnostic score
HLH: Hemophagocytic lymphohistiocytosis
HLH-2004: International HLH diagnostic and therapeutic protocol (2004)
HLH-94: International HLH diagnostic and therapeutic protocol (1994)
ICU: Intensive care unit
IgG: Immunoglobulin G
IU: International unit
IVIG: Intravenous immunoglobulin
MRI: Magnetic resonance imaging
NK: Natural killer
NK-cell: Natural killer cell
PCR: Polymerase chain reaction
PSA: Prostate-specific antigen
QuantiFERON: QuantiFERON-TB Gold test (interferon-γ release assay)
RBC: Red blood cell
sCD25: Soluble interleukin-2 receptor (same as soluble CD25)
TB: Tuberculosis
WBC: White blood cell
